# Supplementary material for: Perceptions, experiences, and beliefs regarding urinary tract infections in patients with neurogenic bladder: A qualitative study
Source: PLoS One. 2023 Nov 1;18(11):e0293743. doi: 10.1371/journal.pone.0293743 (PMC10619779; doi:10.1371/journal.pone.0293743)
Supplement: S1 Fig — (PDF) [file pone.0293743.s001.pdf]

Focus Group Discussion Guide

Focus Group ID:

Date:

Facilitator's welcome, introduction and instructions to participants

Welcome: Hello. My name is XXX and I am a researcher at Edward Hines, Jr. VA Hospital. Thank you for volunteering to take part in this group discussion. You have been asked to participate because you are a Veteran with bladder dysfunction due to chronic neurologic injury or disease and your point of view is important. I realize you are busy, and I appreciate you volunteering your time.

Introduction: This group discussion is specifically designed to talk about your thoughts and feelings about and experiences with urinary tract infections, also called UTIs. Throughout the interview, I'll use the abbreviation UTI to refer to a bladder infection. By your verbal consent, you have agreed to participate in this group discussion. The group discussion will take no more than two hours.

Anonymity: I would like to audio record the discussion to help our research study team remember and subsequently analyze the discussion topics. Despite being audio taped, I want to assure you that the discussion will be anonymous. The tapes will be kept safely in a locked facility until they are transcribed word for word, then they will be destroyed. The transcribed notes will contain no information that would allow individual people to be linked to specific statements. No information discussed in this group will be given to any of your doctors or care team. Do I have permission to audio record this session?

You should try to answer and comment as accurately and truthfully as possible. The other focus group participants and I would appreciate it if you would not discuss comments of other group members outside the focus group. If there are any questions or discussions that you do not wish to answer or participate in, you do not have to do so; however please try to answer and be as involved as possible.

Ground rules

- The most important rule is that only one person speaks at a time. There may be a temptation to jump in when someone is talking but please wait until they have finished.
- There is no need to wait for me to call on you to respond to a question and you do not have to speak in any particular order.
- There are no right or wrong answers
- When you do have something to say, please do so, as long as anyone else speaking has finished their comment. There are many of you in the group and it is important that I obtain the views of each of you. I may call on you, if you are not saying much. Or I may ask you to give others a chance to speak if you have spoken a lot.
- Feel free to respond to another group member, not just to my questions. You do not have to agree with the views of other people in the group but avoid attacking or putting someone else's ideas down.
- Does anyone have any questions? (answers).
- OK, let's begin

Warm up

- First, I'd like everyone to introduce themselves. Please tell us your first name and what type of neurologic injury or disease that you have. If you do not want to describe your neurologic injury or disease, that's OK. Moving forward, please only use everyone's first name.

Introductory question

I am just going to give you a few minutes to think about your experiences with urinary tract infections. Would anyone like to start by sharing his or her experience?

Guiding questions/statements with subsequent follow-up probes

1. Tell me about the last time you had UTI.
    - What symptoms make you think you have a UTI or what are your typical symptoms?
    - What symptoms have you had with UTIs in the past?
2. How worried are you when you think you have a UTI?
    - Do you think your chances of getting a UTI are higher than average?
    - What consequences are you worried about?
3. Tell me what you do when you think you have a UTI. (eg, call the doctor, change catheter, drink fluids)
    - Tell me more about that. How did you decide to do that particular action?
    - What prompts you to see a doctor when you think you have a UTI?
    - If you see your doctor, where do you typically go? (eg, Regular clinic, Urgent Care, ED, etc.)
    - What do you expect to happen at the visit with your doctor?
4. Tell me how your UTIs have been diagnosed in the past.
    - Does the doctor perform a urine test?
    - Has a doctor ever told you that you had a UTI without performing a urine test?
    - Has a doctor ever told you that you did not have a UTI and antibiotics would not be helpful?
        - If so, did that doctor explain to you why?
5. Let's say the doctor says that you probably have a UTI. What do you expect to happen next?
    - If the doctor prescribes you an antibiotic, do you understand why the doctor is prescribing the antibiotic?
    - What do you think will happen if you have a UTI and do not take an antibiotic?
    - Does the doctor suggest any other things to do? (drink fluids, cranberry tablets, changing catheter)
6. Tell me what you know about side effects from antibiotics.
    - Tell me about any side effects you've experienced from taking an antibiotic.
    - Do you think your chances of getting an antibiotic side effect are higher than average?
7. Tell me what you know about preventing UTIs.
    - What information have doctors given you about UTI prevention?
    - What have you tried in the past and did it work?
8. What things are you unable to do when you have a UTI? For example, are you still able to go to work when you have a UTI?
    - What other consequences have happened to you because you had a UTI?
    - What are some medical complications from UTIs that you've experienced?
9. What is the one thing that bothers you the most when you have a UTI?
10. Do you want more education or information about UTIs from your doctor?
    - Do you feel that you have the information you need to understand the diagnosis and treatment of UTIs?
    - Do you usually have questions about UTIs that you have not asked or that have not been answered by the doctor?
    - If you received more education from your doctor about UTIs, how would this help you?
    - What format would you prefer to receive information about UTIs? Written, verbal, handouts, videos? Would it help to also have a caregiver, friend, or family member have education?
    - When would you like to receive this information? (during visit, after visit)

Concluding question

- Please let me know if there are any other final thoughts you would like to share about your experiences with UTIs

Conclusion

- Thank you for participating. This has been a very successful discussion. Your opinions will be a valuable asset to the study
- We hope you have found the discussion interesting
- I would like to remind you that any comments from this discussion will be anonymous
